# Bacteria in Cancer Therapy: Renaissance of an Old Concept

**DOI:** 10.1155/2016/8451728

**Published:** 2016-03-08

**Authors:** Sebastian Felgner, Dino Kocijancic, Michael Frahm, Siegfried Weiss

**Affiliations:** ^1^Department of Molecular Immunology, Helmholtz Centre for Infection Research, 38124 Braunschweig, Germany; ^2^Institute of Immunology, Hannover Medical School, 30625 Hannover, Germany

## Abstract

The rising incidence of cancer cases worldwide generates an urgent need of novel treatment options. Applying bacteria may represent a valuable therapeutic variant that is intensively investigated nowadays. Interestingly, the idea to apply bacteria wittingly or unwittingly dates back to ancient times and was revived in the 19th century mainly by the pioneer William Coley. This review summarizes and compares the results of the past 150 years in bacteria mediated tumor therapy from preclinical to clinical studies. Lessons we have learned from the past provide a solid foundation on which to base future efforts. In this regard, several perspectives are discussed by which bacteria in addition to their intrinsic antitumor effect can be used as vector systems that shuttle therapeutic compounds into the tumor. Strategic solutions like these provide a sound and more apt exploitation of bacteria that may overcome limitations of conventional therapies.

## 1. Introduction

For most patients, cancer diagnosis remains equivalent to suffering, intoxicating therapies and an impending fatal outcome. Very little has changed to this perception over the past fifty years. Despite intensive research as well as improved classical and newly developed therapies, cancer remains the second most frequent cause of death in industrialized societies. Adequate prophylaxis or treatment of other diseases, like cardiovascular disease or infections, has reduced the death toll for these conditions and has dramatically increased life expectancy. However, cancer is essentially a disease of old age. Many molecular alterations are required in the genome of a cell to transform a normal cell into a cancer cell. Thus, the likelihood that such events take place in an individual cell is increasing with age. This also partly explains the rising incidence of cancer. Based on recent official statistics, every second individual will be diagnosed with cancer throughout his life and every fourth will eventually succumb to the disease [1]. This fact illustrates the tremendous impact of cancer on modern society and the need for suitable therapies that can cure it.

Conventional therapies like surgery, radiotherapy, or chemotherapy remain the backbone of cancer therapy to date. However, not every cancerous tissue can be targeted with a scalpel and physical and chemical methods in general do not distinguish between healthy and malignant tissues [2, 3]. In order to overcome these drawbacks, extensive research was carried out to widen the knowledge on carcinogenesis. This revealed cancer as a highly complex and multifactorial type of disease [4, 5]. Hanahan et al. have conveniently summarized this research and defined ten particular characteristics, termed “hallmarks” of cancer, which equally highlight potential starting points for novel therapies [6, 7].

In this context, immune surveillance is now generally accepted as the hosts own defense system against the development of cancer. However, tumors may become resistant to immune destruction through mechanisms of immune editing or Darwinian selection [8, 9]. Thus, under such circumstances a “mutator phenotype” may prevail which not only counterbalances immune surveillance but also overturns it. Physiological consequences of this imbalance may lead to a tumor microenvironment of “repair” rather than “destruction” [9]. Active cancer immunotherapy aims to reverse this imbalance through compensatory immune activity. The ultimate aim of such therapies is to achieve not only transient effects but also long lasting antitumor effects through engaged immunological memory. After years of research and many set-backs, some cancer immunotherapies have become available in the clinics [10–13]. Therefore, Science magazine selected cancer immunotherapy as the scientific breakthrough of the year in 2013.

Considering all the efforts and difficulties in this therapeutic development before reaching sound therapeutic candidates, it is surprising that the first systematically applied cancer immunotherapy used in more than a thousand patients dates back to a time where radiotherapy and chemotherapy had not yet been invented.

## 2. William Coley: Lessons Learned from the Past

The idea of using bacteria in cancer therapy is often presented as a new approach but in reality predates that of conventional therapies by a long run. The first report describing a connection between infections and a treatment of so-called swellings (most likely cancerous tissue) was documented by the Egyptian physician Imhotep 2600 BC [14]. During the course of history, similar examples can be found. More recently, in 1813, tumor retardation was also observed in cancer patients suffering from gas gangrene, nowadays known to be caused by* Clostridia* sp. [15]. Likely intrigued by historical examples, the first “clinical trial” was performed by W. Busch and colleagues in Berlin in 1863. They intentionally infected a female cancer patient by transferring her into a contaminated bed in which a patient had died from Erysipelas, an infection caused by* Streptococcus pyogenes*. As expected, the patient became infected. As a result, the tumor did regress; however, also the patient died from the infection [16, 17]. The inability to control bacterial infections at that time was limiting its general clinical application.

Inspired by history and uncontrolled trials, the American physician William Coley (1862–1936) became the first pioneer of cancer immunotherapy. Although Coley also started with live bacteria, capable of killing his patients, he was the first to notice that an accurate balance between the therapeutic benefit and infection control was essential for feasibility of this type of treatment. As a solution, he applied a mixture of heat-inactivated* Streptococcus pyogenes* and* Serratia marcescens*, known as “Coley's toxin,” to many patients afflicted with inoperable sarcomas. The treatment was very effective. During his career, he applied his “toxin” to more than 1000 cancer patients. Whenever possible, he injected it directly into, or in close proximity to, the primary tumor mass and kept increasing the dose over the course of therapy [17–19]. As a common side effect, he observed episodes of fever. According to his records, a lengthy treatment and onset of fever were correlating with the success of the therapy [20–22]. Tumor regression was observed in many cases and for some patients even complete clearance of the primary tumor mass and a disease-free state were obtained [20].

Although Coley's work was met with certain success at the time, his work was not acknowledged and rather forgotten until the approach had been approved by the American Medical Association in 1936, after the death of W. Coley. A possible reason for this development might have been the inability to explain the therapeutic mechanism and to control bacterial infections or side effects of the therapy. At the same time, the possibility of radiotherapy was emerging and providing a competitive alternative to bacteria mediated cancer therapy. Prohibiting advancement of Coley's therapy was certainly also the standpoints of leading US cancer pathologists who strongly opposed Coley's therapeutic approach [20]. Although bacteria mediated tumor therapy was still subject to investigation after Coley's death, it had been marginalized for decades. In the 1960s and 1970s, small clinical trials were performed with formulations similar to Coley's toxin (e.g., Vaccineurin) but the results were variable. Furthermore, a controlled study performed in 1962 showed a therapeutic response in 20 out of 93 cancer patients indicating a difference in individual susceptibility to this kind of therapy [23]. However, it is important to note that a comparison between Coley's therapy and later attempts should take into consideration differences in the specific formulation, the course of treatment including duration and frequency, and prior or simultaneous application of chemotherapeutic and antipyretic drugs [24].

Nevertheless, bacteria can be found in the clinics as routine treatment for cancer patients nowadays. Since the late 1980s, oncologists were successfully using the vaccine variant of* Mycobacterium bovis* BCG (Bacille Calmette-Guerin) as agent to prevent relapses of bladder cancer after surgical removal of the primary tumor [25]. Although the exact mode of action of the bacteria is not fully understood, they might enhance the immune response against the cancer cells by, for example, activation of natural killer cells [26–29]. Also the idea to use bacteria systemically is undergoing renaissance. The progress in biomedical research as well as the increasing knowledge on bacterial behavior and genetic engineering is most likely the reason for the enhanced attention on bacterial tumor therapy.

## 3. Current Concepts of Bacteria Mediated Cancer Therapy

Using animal models, several Gram-positive and Gram-negative bacteria of various genera including* Clostridia* sp.,* Salmonella* sp.,* Bifidobacteria* sp., and* Listeria* sp. have already been tested for their potential in cancer therapy.  Table 1 summarizes former and recent results of representative studies dealing with bacterial therapies.

In the last half of the 20th century, obligate anaerobic bacteria like* Clostridia* spores were preferentially used for cancer therapy. Their spores germinate only in the absence of oxygen. Therefore, these bacteria are specific for necrotic areas, a frequent and unique characteristic of solid tumors [30–33]. Thus, they should provide a high safety profile in patients. Although germination of* Clostridia* was indeed confined to hypoxic regions, toxicity conferred by exotoxins led to high mortality rates using this type of bacteria. To increase the safety of* Clostridia*, virulence factors like the *α*-toxin had been deleted from potential therapeutic strains. Aside from experimental studies in mice,* C. novyi-*NT (nontoxic) has already been tested in preclinical and clinic trials using dogs as well as human patients (see Table 2). Results were promising and the advanced leiomyosarcoma of a human patient was positively affected upon intratumoral injection of* Clostridia* spores [34]. Furthermore, in a recently published report, orthotopic glioblastomas were successfully targeted with* C. novyi* spores upon intravenous infection in a rat model [35]. These data indicate that the spores are able to pass the blood brain barrier under certain conditions. Although the mechanism of the antitumor effect by* Clostridia* is poorly understood, these bacteria are able to successfully target neoplastic tissue without seriously harming the host. Therefore, they might represent a suitable carrier in bacteria mediated tumor therapy. However, the restriction to anaerobic regions might also be a great disadvantage of* Clostridia*. Metastases or small tumors without necrotic areas as colonizing niche may not be targeted as easily by this therapeutic approach.

To overcome the limitation of confinement to hypoxic regions and to address the problem that tumors outgrow from viable oxygenated tissue, facultative anaerobic bacteria like* Salmonella* Typhimurium moved into focus of bacteria mediated cancer therapy. However, as* Salmonella* are able to grow under aerobic conditions, they are not restricted to only colonizing tumors but are also able to disseminate to healthy organs like spleen and liver [36]. To ensure safe application,* Salmonella* need to be adapted. Coley already realized that a level of control is needed for the success of his therapy. As he had observed for* S*.* pyogenes,* wild-type bacteria are efficiently capable of attacking cancerous tissue, however not without compromising patients' safety. In an attempt to solve this problem, he adhered to heat inactivation, albeit noticing a decrease in therapeutic efficacy [18]. Rather than crude heat inactivation, later attempts have sought to reinstall safety via means of attenuation by genetic alteration.

Nowadays, suitable strains have been generated. They are obtained either by* in vitro* or* in vivo* passaging or by targeted gene deletion using molecular techniques [37–40]. The first technique relies on the selective pressure when the bacteria adapt to a particular environment within the host. By passaging bacteria from tumor to tumor either in cell culture or in mice, they can develop a tumor adapted phenotype concurrently exhibiting high tumor specificity. The prominent* Salmonella* strains VNP20009 (*in vitro*) and A1-R (*in vitro* and* in vivo*) emerged from this type of strategy [41–43]. Auxotrophy for purines or Arg and Leu, respectively, rendered these* Salmonella* variants metabolically deficient and increased tumor specificity. Particularly, the strain A1-R was shown to be highly effective in various cancer models [44–51]. However, the random introduction of certain deletions or single point mutations through selective pressure represents an uncontrollable way to properly tailor bacterial strains. It was reported that VNP20009 was coincidentally bearing a deletion of 120 genes that might affect its* in vivo* performance [52]. In addition, it contains a SNP in the chemotaxis gene* cheZ*. As consequence, the whole flagella synthesis is impaired. The strain was therefore devoid of a potent immune-stimulating factor. Decreased immunogenicity may provide one explanation for the poor outcome of the clinical trial performed with this strain in 2001 [53]. Due to the uncertainties affiliated with such an unspecific method of attenuation illustrated by the previous examples, targeted gene deletion may be a better choice for tailoring bacteria for cancer therapy.

**Figure 1:**
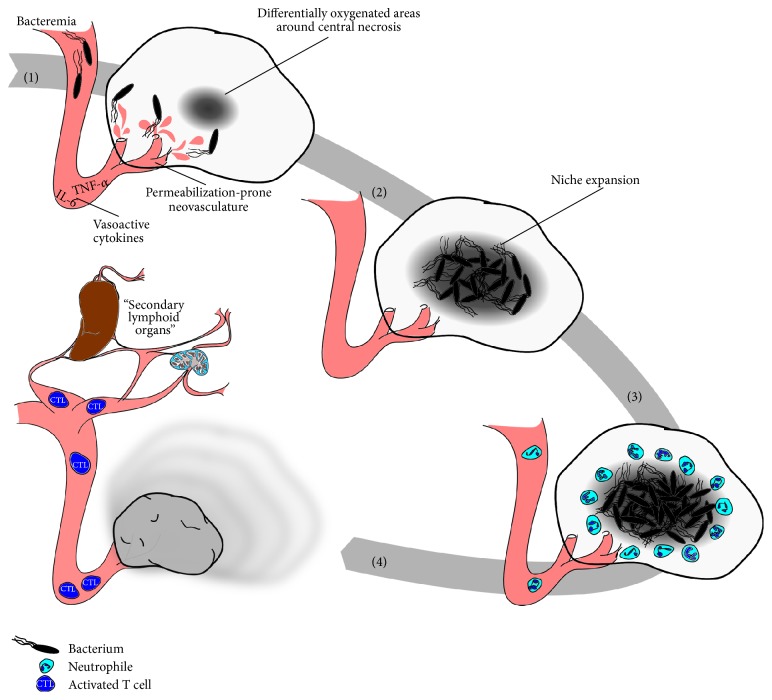
Biological events of the intrinsic tumor therapeutic effect of* Salmonella*. Steps  (1)–(4) depict major biological events associated with an antitumor effect induced by* Salmonella* infection. (1) Tumor invasion. (2) Colonization. (3) Infection control. (4) Antitumor response and tumor regression. (1) Presence of bacteria in the blood stream (bacteremia) induces a cytokine storm, which is dominated by vasoactive cytokines that facilitate passive deposition of bacteria in the tumor during the induced hemorrhage. (2) Invading bacteria accumulate, localize to a preferable growth environment (low p0_2_, immune privileged site), and proliferate to saturation. (3) Colonization brings about change to the tumor microenvironment, including attraction and polarization of innate effector cells and cytokines in favor of antibacterial control and tumor immune surveillance. (4) Tumor regression occurs in response to multimodal therapeutic effects, including an adjuvant effect on preestablished tumor immune surveillance, polarization of innate effectors' phenotype, direct cytotoxicity, and passive effects.

The intrinsic antitumor response of bacteria is most likely connected to their microbial associated molecular patterns (MAMPs) like LPS, flagella, and CpG [54–57]. Some of these molecules represent suitable targets for modification. However, such modifications can easily lead to overattenuation that might increase their safety but at the same time compromise their ability to induce a sufficient antitumor response. Nevertheless, the trial and error concept of finding proper bacterial variants of* Salmonella* that can safely stimulate immunity against the tumor was leading to the discovery of the aforementioned candidates VNP20009 and A1R which have already been tested in various preclinical setups and/or clinical trials. Even though the antitumor response induced by* Salmonella* is partially understood nowadays (Figure 1), designing them properly for therapy remains challenging [58]. As seen in Figure 1, a strong induction of the innate immune system, for example, high levels of TNF-*α*, is initiated by* Salmonella* to promote colonization of solid tumors [59, 60]. This is accompanied by a* Salmonella*-induced adjuvant effect concurrently activating the adaptive immune response. As consequence, tumor-specific cytotoxic T lymphocytes (CTL) that are able to destroy the remaining cancerous tissue have been found to increase in numbers [61].

## 4. Clinical Trials: Lessons Learned for the Future

Prominent representatives of the genera* Listeria*,* Clostridia*, and* Salmonella* have made it into clinical trials. However, while* Listeria monocytogenes* (ANZ-100) was primarily tested as a recombinant attenuated therapeutic live vaccine for advanced cancer patients [62–64], the trials with* Clostridia* and* Salmonella* relied predominantly on the intrinsic antitumor effect of the bacteria (Table 2).

For instance, in 1947, the first bacterial filtrates of* C. histolyticum* spores entered clinical trials [65]. Although preferential accumulation in the cancerous tissue along with antitumor effects was observed, the systemic toxicity induced by exotoxins released from the bacteria was too high. Furthermore, the inefficiency of* Clostridia* spores in normoxic areas most likely facilitated tumor regrowth [66]. As consequence, recent clinical trials were performed with nonpathogenic* Clostridia* sp. such as* Clostridia butyricu*m M-55 or* Clostridia novyi*-NT (Table 2). In 2012, six client-owned dogs, nonresponsive to standard therapy, were treated with* C. novyi*-NT. Again, the spores preferentially germinated in the tumor but were still able to induce some acute toxic effects including fever, nausea, and diarrhea [67]. Despite these side effects, the authors suggested to carry on with clinical trials [66]. Accordingly, Roberts and colleagues treated a human patient with an advanced leiomyosarcoma and observed ongoing tumor regression after several intratumoral applications of spores [34]. We believe that this will serve as a basis for further clinical trials. While it is now established that the antitumor effect of* Salmonella* relies on an induced anticancer immune response [59, 68], the therapeutic mechanisms of* Clostridia* have not yet been studied in detail. Considering the local effects of germination during infection, the response might be mediated by direct histolytic activities. In accordance, the mechanism of tumor invasion, the mode of action, and the role of the immune system need to be investigated in order to optimize the cancer therapy with spores of* Clostridia*.

In case of* Salmonella*, the highly attenuated strain VNP20009 was tested in humans and dogs in 2002 and 2005, respectively [53, 69]. This strain had been specifically designed for bacterial cancer therapy. It exhibits a* purI* ^−^ *xyl* ^−^ genotype and was shown to be hyperinvasive in human M2 melanoma cells [70]. Additionally,* msbB* gene had been deleted to render Lipid A molecule less immunogenic [71, 72]. While this strain was shown to colonize murine tumors efficiently and exhibit strong antitumor effects, the results in canine and human hosts were not as prominent [53, 69]. In dogs, colonization in only 42% of subjects was accompanied by a response rate of 25%, while colonization and therapeutic response in the human environment almost failed completely. This comparison illustrates the translational challenge in turning from one host to the other, that is, from transplantable tumors in mice to the application in humans.

Bacteria are known to be more resistant towards murine effector mechanisms. In support, the human trial did show that most of the bacteria were cleared from the circulation within 60 min. This might suggest a distinguishing role of complement lysis and phagocytic clearance upon systemic application to human patients. Such are most important aspects to consider for a successful cancer therapy using bacteria [54]. Furthermore, mice were usually kept under specific pathogen-free conditions and had not been preexposed to* Salmonella* Typhimurium prior to the therapy. In contrast, some humans and dogs may have been preexposed and therefore already possess immunity to the bacteria to a certain level [73]. Moreover, one also needs to carefully consider the pretreated state of the patients in these particular trials. Human patients were often preexposed to chemotherapeutics, which may per se impact the immune system and thus reduce its responsive capacity. Along the same line, a discrepancy may arise from a cohort selection of late-stage cancer patients that may already have been highly immune compromised. In particular a therapy with a mode of action that predominantly relies on the activation of an immune response might crumble under these preconditions. One possible explanation why Coley was so successful may be that he deployed his mixture as first-line treatment or after surgical resection [18].

Reviewing Coley's work, another important aspect should be discussed; namely, does tumor colonization constitute a prerequisite for a therapeutic effect or is the whole process sufficiently initiated by the bacterial induced cytokine storm following bacteremia? Coley was using heat-inactivated bacteria which are unable to establish colonization of solid tumors. Regardless, antitumor activity was still observed in most of his cases. It was suggested that streptokinase of* S. pyogenes* might constitute an effector molecule of Coley's toxin [74]. However, heat inactivation would have also affected production and secretion of this particular molecule. Nowadays, it is known that bacteria express so-called MAMPs which are recognized by pattern recognition receptors and are to a large extent heat stable. They stimulate cells of the immune system which are at least partly responsible for the therapeutic activity. In accordance, mice that either are lacking the Toll-like receptor 4 (TLR4) or are defective in MyD88 signaling did not display any antitumor response upon administration of* Salmonella* [75, 76]. Along this line, single injection of LPS alone has been shown in particular tumor models to elicit strong antitumor effects in Wt mice and induce specific tumor responses [59]. Therefore, tumor colonization might not be an essential driving force, but rather the MAMPs provided may act as adjuvant for priming or activating the immune system at inductive lymphoid organs. This, however, remains speculative. More work is needed to elucidate the intermittent relationship between traits of bacterial infection and an antitumor effect. Nevertheless, currently various purified or synthetic MAMPs are evaluated in preclinical and clinical trials (reviewed in [77]).

In the 2001 clinical trial, the VNP20009 strain of* Salmonella* was administered to cancer patients with metastatic melanoma. In the vast majority of cases, with a few exceptions, VNP20009 failed to colonize tumors. Hence, any antitumor response must have been initiated by MAMPs supplied from sites extrinsic to the tumor. However, also no significant antitumor effects were observed among the patient cohort in this particular study. Studies in mice have shown that while some highly immunogenic tumors like the colon carcinoma CT26 can be easily affected with systemically applied purified LPS or dead bacteria, more resilient tumors like RenCa (a renal adenocarcinoma) may not be similarly affected by the same treatment [54]. Therefore, the efficacy of a MAMP-based therapy depends not only on the potency of the bacterial infection but also on the immunogenicity of the tumor or the effectiveness of its escape mechanisms.

Explanations for the failure of VNP20009 may be numerous. On one hand, the previously mentioned influence of prior chemotherapeutic treatment on immune cells may have had an impact on the efficacy of the therapy. On the other hand, the strain may have been simply overattenuated for the human immune system, meaning that VNP20009 was probably cleared long before it was able to induce a proper immune response or establish tumor colonization. In particular, the aforementioned large gene deletion and the abolished flagellar synthesis could have had dramatic effects on the fitness and immunogenicity of the bacterial strain (e.g., by its inability to trigger TLR5 via flagellar PAMPs [78]). Based on these considerations a new concept of strain design has to be defined to appropriately enhance the immunogenicity of a mutant strain while retaining its attenuated character.

## 5. Optimal Tumor Targeting* Salmonella* Strains: Requirements and Promising Strategies

**Figure 2:**
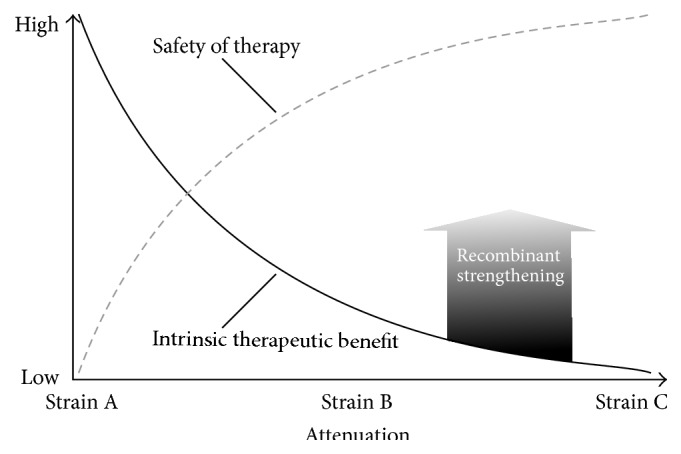
Hypothetical relationship between intrinsic therapeutic benefit and safety using* Salmonella* for bacteria mediated tumor therapy. Empirical observations support an inverse relationship between intrinsic therapeutic benefit and safety of treatment associated with* Salmonella*-based tumor therapy. Strain variants A–C in order of increased attenuation. Depicted are three scenarios, each intrinsically inadequate for achieving therapeutic success. Reinstalling/complementing an intrinsic therapeutic effect may be accomplished via recombinant strengthening without affecting the aforementioned intrinsic relationship.

Interestingly, the lessons learned from recent clinical trials are similar to those Coley made nearly hundred years ago. Although the technology was not available to genetically modify microorganisms like* Streptococcus* at that time, Coley's heat inactivation approach generated a proper balance between therapeutic benefit and safety nevertheless (Figure 2). At the very least, one may have deemed it a success under the set of conditions he was facing regarding patient status, that is, no chemotherapy and first line therapy. Nowadays, the requirements are different and genetic engineering is essential to tailor bacteria for cancer therapy.

**Figure 3:**
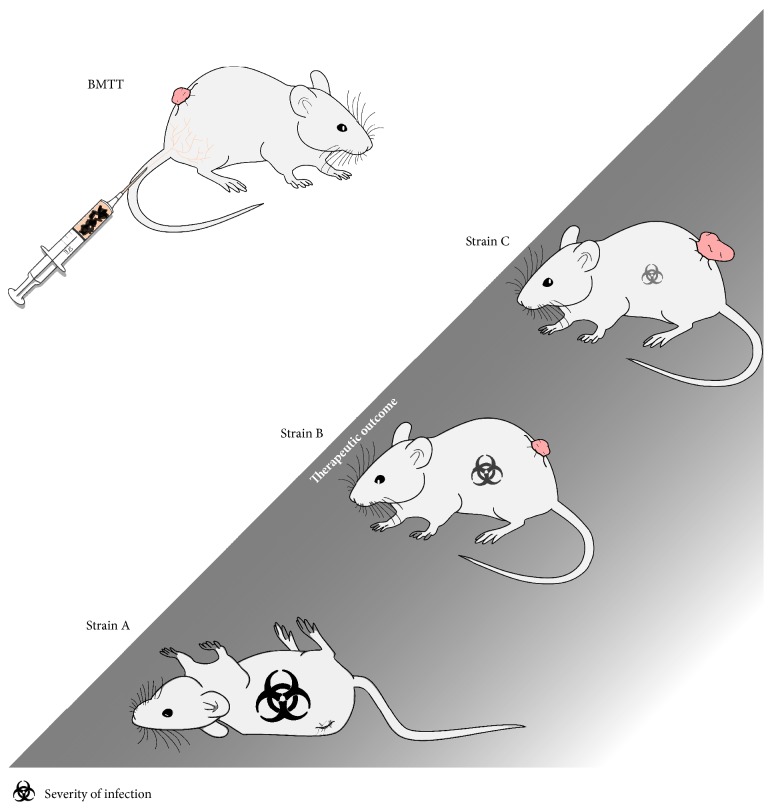
Various phenotypic outcomes in mice following treatment with* Salmonella*. The inverse relationship between intrinsic therapeutic benefit and safety of bacteria mediated tumor therapy (BMTT) using* Salmonella* can be demonstrated through manifestation and progression of cancer and infection in mice. An inadequately attenuated, thus virulent, strain may successfully retard/regress tumor development albeit concurrently resulting in morbidity and mortality of the patient (strain A). Conversely, overattenuation may ensure patient safety at the cost of therapeutic efficacy (strain C). Intermediate levels of attenuation may yield corresponding levels of cancer progression and manifestation of infection (strain B). Thus, developing a properly balanced strain remains a challenge for successful therapy using* Salmonella*.

Again, it is important to emphasize that for therapeutic success an ideal balance between immune stimulation and attenuation has to be determined and reached by strain design (Figure 3). Previous studies have shown that single gene deletions are sufficiently able to attenuate* Salmonella* in a murine environment [54, 60, 71, 79–81]. Several of these deletion strategies either interfere with virulence factors (i.e., important MAMPs) or restrict the* in vivo* survival of the bacteria by metabolic auxotrophies [82, 83]. However, as mentioned in the previous section, only focusing on rendering a bacterium safe for administration might not be the proper solution to achieve a suitable strain for cancer therapy. Take, for example,* Salmonella* variants that lack an essential parts of the LPS molecule (e.g., Δ*rfaD*). Mice are symptomless upon infection with this particular mutant strain. However, drastically reduced antitumor effects were observed [54]. The conclusion is that the strain had been overattenuated. Based on these findings, it is not advisable to shut down bacterial virulence factors that are obviously important for inducing the essential immune response for therapeutic effects. Hence, a bacterial strain has to be designed by genetic engineering in a way that the microorganism is both attenuated and optimized at the same time. Along these lines, several interesting approaches have been tested for vaccination purposes in the past few years that could potentially be transferred to cancer therapy [38].

One approach is to generally increase the adjuvant effect of bacteria. The immune recognition of* Salmonella* and induction of an immune response directly correlate with the presence of various MAMPs. To survive in a hostile environment* Salmonella* may either modify the structure of the MAMP or downregulate the expression of certain immunogenic factors like flagella [72, 84–86]. To counteract such mechanisms, a promising recombinant strategy would be to reinstall the immunogenicity of* Salmonella* via modification of immunogenic targets/MAMPs. For example, a hexa-acylated Lipid A structure was shown to be highly efficient at stimulating TLR4, while tetra-acylated Lipid A acts as an antagonist [87]. Therefore, a mutant (Δ*pagP *Δ*pagL *Δ*lpxR*) only expressing the hexa-acylated Lipid A structure was shown to enhance the therapeutic effect [72, 88]. In addition, it was shown that* Salmonella* variants bearing both flagella proteins FliC and FljB trigger an increased host immune response upon oral administration [89, 90]. These examples demonstrate that the immunogenicity of attenuated bacteria can be enhanced when the MAMPs are modified in a way that host pattern recognition receptors (PRR) are more efficiently stimulated.

However, modifying the expression of certain MAMPs could have pleiotropic effects, some detrimental, that may affect the regulatory circuits of bacteria in a more general way. Therefore, a wild-type like phenotype of bacteria that is only conditionally modified may be the next step in strain design. Currently, two concepts are evaluated using this rationale, namely, delayed attenuation and delayed lysis [38, 54, 91–96]. Such mutants exhibit a wild-type like phenotype upon* in vivo* administration while carrying a modified genotype. For instance, auxotrophic/attenuated bacteria may express a complementing gene under an inducible promoter like P_BAD_ or P_tet_. Their activation depends on presence of arabinose or anhydrotetracycline, respectively. Such bacteria can be inducibly complemented in culture.* In vivo*, the inducers are diluted out and no longer available. As consequence, the bacteria will lose their WT phenotype and become attenuated after a few rounds of replication. This delayed attenuation system was recently deployed for* Salmonella* to modify the LPS structure under the control of P_BAD_. The effect was evaluated in a murine tumor model [54]. The wild-type like phenotype of the administered bacteria induced a strong immune response that significantly enhanced the antitumor response compared to the bacteria carrying the gene deletion only. None of the mice succumbed to the infection and the health status of the mice was only transiently affected after bacterial administration [54].

Similarly, in the delayed lysis system cell wall synthesis is abrogated in the absence of arabinose* in vivo* [97]. The bacteria are thus not able to establish a systemic infection. However, the sudden cell death* in vivo* might cause complications like toxic shock due to release of large amounts of endotoxin and other PAMPs. Nevertheless, the system was successfully tested to vaccinate mice against influenza viruses, by a targeted release of intracellular virus specific antigens by the bacteria [98]. The system may be no less useful in a cancer model and should thus be explored.

These results demonstrate that modern strategies may accommodate attenuation and optimization in the same therapeutic strain. Nevertheless, many tumors still remain completely resistant to such a therapy or might only be delayed in growth. The technological progress has now fostered the possibility to modify bacteria not only to the needs of safety but also to the needs of efficacy. The possibility of simply focusing on intrinsic therapeutic effects has been challenged. In this regard, combining chemotherapeutic approaches together with bacteria may represent a more realistic and promising strategy for ongoing research efforts [48, 99, 100]. In particular, the combination with checkpoint inhibitors like *α*-PDL1 or *α*-CTLA4 has shown increased efficacy [101–103]. However, in this review, we restrict our focus to the strategy of W. Coley and the possibilities in recombinant strengthening of tumor targeting bacteria. Bacteria could be designed to shuttle therapeutic compounds like chemotherapeutic drugs directly into the cancerous tissue. This should maximize their effect while reducing systemic side effects. Bacteria with their intrinsic tumor specificity provide a unique tool kit for this application.

## 6. Bacteria as Tumor Targeting Shuttles of Therapeutic Molecules

Exploiting bacteria as live vector systems could represent the next generation of strain design. However, this promising idea is affiliated with its own challenges [104]. A tumor-specific bacterium and a compound that can be synthesized and, if necessary, actively secreted by the bacteria are both required.

Two concepts are currently under investigations. The first one employs prodrug converting enzymes produced by bacteria. This strategy relies on enzymes that are capable of converting a systemically administered inactive prodrug into an active cytotoxic “drug.” As the enzyme would be present only in vicinity of the bacteria and facilitate conversion at only this site, this therapy requires high tumor specificity to ensure local efficacy and activity only against the target tumor. The therapeutic benefit of enzymes like cytosine deaminase and nitroreductase expressed by either* Clostridia* or* Listeria* has been tested [105–110]. However, while they showed promising activity* in vitro*, no significant improvement of therapeutic effects was observed* in vivo*. Most likely, the expression levels or the efficacy of secretion or conversion was too low inside the cancerous environment and needs to be optimized for further attempts. However,* Salmonella* as carrier of enzymes like cytosine deaminase, thymidine kinase, or CPG2 have been successfully used in preclinical and clinical setups [70, 107, 111]. Nevertheless, the cohort of patients needs to be enlarged to significantly confirm the promising results.

The second approach concerns production and subsequent secretion or release of therapeutically active compounds by the bacteria themselves during tumor colonization. Therapeutic molecules include bacterial toxins like *α*-Hemolysin or Azurin [112, 113], recombinant effector proteins such as mTNF-*α* and rIL-2 [114], or small hairpin RNAs (shRNAs) [115–117]. The delivery of these molecules across bacterial membranes to the extracellular environment represents a major challenge, although not in focus in many studies as long as beneficial effects are obtained. Most of such constructs do not contain obvious signals known to be required for excretion by* Salmonella*. Most likely, in these cases, the bacteria passively release the compounds upon lysis. Furthermore, despite the impressive therapeutic effect [117], it remains unclear whether the production of the effector proteins or shRNA is essentially required within the tumor or whether it is sufficient to deliver them in the periphery. To answer these questions, more studies should be performed. Nevertheless, most of the* in vivo* studies described thus far are held back by a lack of efficacy due to inefficient delivery of the compounds. Therefore, optimizations that would ensure controlled delivery may be of great advantage to increase therapeutic efficacy.

Two promising active delivery strategies for Gram-negative bacteria like* Salmonella* are currently under investigation. The first includes the aforementioned delayed lysis system. The advantage would be the controlled release of a therapeutic compound with a high concentration in a short period of time. However, it is a one-point release system and does not allow for continuous expression. Using this strategy, stimulation of the immune system may be too short-lived or the degradation of the compound may be too fast to treat the tumor in a sustained manner. Nevertheless, as the system has been successfully applied for vaccination purposes, its potential in cancer therapy should be explored as well.

The second strategy aims to exploit secretion systems that are already present in every bacterium [118, 119]. The basic idea is to fuse therapeutic compounds to signaling molecules that are essential for delivery via a particular secretion system. Thus the compounds would be continuously secreted. In order to obtain high tumor specificity, the application of inducible or tumor-specific promoters [54, 83, 120–125] may be preferred. However, the fusion to a signaling peptide could be a limiting factor to this strategy. The compound may lose its activity due to, for example, conformational alteration or incorrect refolding upon secretion. However, proof of principle has been successfully demonstrated by Singer et al., where recombinant neuroactive peptides were delivered via the flagellar type three secretion system (fT3SS) [126]. Furthermore, the efficacy of T3SS for delivery was assessed in a cancer vaccine, where the codon-optimized human tumor-associated antigen Survivin was genetically fused to* sseJ*. As a result, complete tumor regression was observed [127]. Thus, delivery via the T3SS of* Salmonella* may represent a promising foundation for active delivery.

Taken together, the possibility to deliver therapeutic compounds actively and directly to the site of interest may be the greatest advantage of bacteria and therefore it is worth placing further focus on this therapeutic application of bacteria. Results are promising and highlight with confidence a sustainable and opportune direction in bacteria mediated tumor therapy.

## 7. Conclusion

The specificity of cancer therapy for neoplastic tissue remains the most desirable trait. While many different strategies nowadays struggle to generate a sustainable targeted treatment, conventional therapies still remain the backbone of cancer remedies. However, they distinguish cancerous tissue from healthy tissue only by an increased proliferation rate. Therefore, room for improvement to such traditional therapies is evident. Furthermore, novel targeted therapies like the use of monoclonal antibodies or cell mediated immunotherapy are halted by applicability and costs and may yield therapeutic solutions to only a fraction of patients. With the need for a better general differentiator for cancer therapy, the great potential in tumor specificity that is intrinsic to bacteria should be exploited more readily not only for therapy but also for diagnosis as shown recently [128]. Focusing on the immunogenic therapeutic effect of bacteria alone might be insufficient. Intelligent exploitation of the unique tumor colonizing property for drug delivery could provide the next step needed to substantiate a more senseful and sustainable bacterial mediated tumor therapy that may pave the way to routine application in the clinics.

## Figures and Tables

**Table 1:** Recent representative preclinical examples of bacteria mediated tumor therapy (BMTT) *in vivo *from 2012 onwards.

Species	Year	Model	Result	References
*C. novyi*	2014	Spontaneous, dog	Colonization and prolonged survival	[34]
*C. novyi*	2015	Glioblastoma, rat	Colonization and prolonged survival	[35]
*B. infantis*	2013	Bladder, rat	High tumor specificity by engineered strain	[129]
*L. monocytogenes*	2014	Ovary, mouse	Reprogramming of M2-M*ϕ*, iNOS-mediated tumor cell lysis	[130]
*L. monocytogenes* (ANZ-100)	2014	Pancreas, mouse	Prolonged survival	[131]
*S. *Typhimurium (A1-R)	2012	Breast, nude mouse	Intravenous administration most effective for tumor targeting	[132]
*S. *Typhimurium (A1-R)	2012	Brain, nude mouse	Tumor growth inhibition and prolonged survival	[133]
*S. *Typhimurium (A1-R)	2014	Bone metastasis, nude mouse	Inhibition of breast cancer bone metastasis	[47]
*S. *Typhimurium (A1-R)	2014	Pancreas, nude mouse	Tumor growth retardation, Α-VEGF supports therapy	[134]
*S. *Typhimurium (A1-R)	2015	Ovary, nude mouse	Prolonged survival, less metastasis formation	[51]
*S. *Typhimurium	2015	Carcinoma (CT26), mouse	Complete tumor rejection in all cases by recombinant strain	[54]
*E. coli*	2015	Breast (4T1), mouse	Secretion of toxic compound, reduced tumor volume	[113]
*La. acidophilus*	2014	Carcinoma (CT26), mouse	Increased apoptosis and tumor growth suppression	[135]

*C. = Clostridium; B. = Bifidobacterium; L. = Listeria; S. = Salmonella; La. = Lactobacillus; E. = Escherichia*.

**Table 2:** Recent examples of clinical trials with bacteria mediated tumor therapy (BMTT) since 2002.

Species	Year	Cohort	Result	References
*S. *Typhimurium (VNP20009)	2002	24 patients with metastatic melanoma; 1 patient with metastatic renal cell carcinoma	Induction of immune response, tumor colonization in 3 cases, no antitumor response	[53]
*S. *Typhimurium (TAPET-CD)	2003	3 patients with advanced and metastatic solid tumors	66% tumor colonization, measurable activity of cytosine deaminase in tumor	[107]
*C. novyi-NT*	2014	1 patient with advanced leiomyosarcoma	Tumor reduction within and surrounding the bone	[34]
*C. novyi-NT*	Active	Patients with solid tumors that do not respond to standard therapy	Recruitment (NCT01924689)	—
*L. monocytogenes* (ANZ-100 and CRS-207)	2011	26 patients with solid tumors (liver, pancreas, lung, or ovary)	Safe vaccines that resulted in immune activation	[64]
*L. monocytogenes* (CRS-207)	Active	90 patients with pancreatic cancer	Extended survival with minimal toxicity	[136]

*C. = Clostridium; L. = Listeria; S. = Salmonella*.

## Additional Points

This review compares historical attempts with present ones of BMTT in order to give perspectives for future studies. It was not the intent to give a comprehensive review of all aspects present concerning BMTT. Therefore, we apologize to our colleagues whose work was not cited here. Note also that the title of this paper includes Renaissance of an Old Concept. Of course, this concept, born over 100 years ago, has received, and will most likely continue to receive, critical scrutiny.
